# Perceptions of Digital Technology Experiences and Development Among Family Caregivers and Technology Researchers: Qualitative Study

**DOI:** 10.2196/19967

**Published:** 2022-01-28

**Authors:** Chen Xiong, Andrea D'Souza, Graziella El-Khechen-Richandi, Alex Mihailidis, Jill I Cameron, Arlene Astell, Emily Nalder, Angela Colantonio

**Affiliations:** 1 Rehabilitation Sciences Institute University of Toronto Toronto, ON Canada; 2 Toronto Rehabilitation Institute-University Health Network Toronto, ON Canada; 3 Acquired Brain Injury Research Lab University of Toronto Toronto, ON Canada; 4 Department of Occupational Science & Occupational Therapy University of Toronto Toronto, ON Canada

**Keywords:** caregiving, digital technologies, sex, gender, diversity

## Abstract

**Background:**

Caregiving is highly stressful and is associated with poor mental and physical health. Various technologies, including mobile and eHealth apps, have been developed to address caregiver needs. However, there is still a paucity of research examining the technology perceptions of informal caregivers, especially from the perspectives of sex, gender, and diversity.

**Objective:**

To address the research gap and inform the development of future caregiving technologies, this study aims to examine how family caregivers perceive using technology to assist with their caregiving routines; identify the sex, gender, and diversity factors that shape these perceptions; and understand how these perceptions and needs are reflected within the current technology development process.

**Methods:**

Semistructured interviews were conducted with 16 informal caregivers of individuals with a range of chronic medical conditions and 8 technology researchers involved in caregiving technology projects.

**Results:**

Three main themes with subthemes were developed. The first main theme is that *caregivers see a need for technology in their lives,* and it comprises the following 3 subthemes: *caregiving is a challenging endeavor*, *technology is multifaceted*, and *caregiver preferences facilitate technology use.* The second main theme is that *relationships play a vital role in mediating technology uptake,* and it comprises the following 2 subthemes: *the caregiver-care recipient dynamic shapes technology perceptions* and *caregivers rely on external sources for technology information.* Finally, the third main theme is that *barriers are present in the use and adoption of technology*, and it comprises the following 2 subthemes: *technology may not be compatible with personal values and abilities* and *technology that is not tailored toward caregivers lacks adoption.*

**Conclusions:**

The findings highlight the multifaceted role that technology can play in aiding caregiving while drawing attention to the perceived drawbacks of these technologies among caregivers. The inclusion of technology researchers in this study provides a more holistic understanding of technologies in caregiving from their initial development to their eventual uptake by caregivers.

## Introduction

Globally, an increasing number of individuals are providing unpaid assistance and support to family members or acquaintances with physical, psychological, or developmental needs [1]. Approximately 17% of Americans have provided care to adults who are ill, disabled, or aged [2]. In Canada, approximately 25% of Canadians aged 15 years and above provide help or care to a family or friend with a chronic health problem [3], whereas in the United Kingdom, approximately 10% of the population are family caregivers [4]. As families and households are getting smaller because of lower birth and marriage rates, there are fewer family caregivers to meet the increasing care demands of the growing older population [5]. This creates challenges in maintaining a work-caregiving balance, assisting with daily living activities, and managing services for the care recipient [2,6]. As such, caregiving remains a stressful experience and exerts a considerable burden on family caregivers [7-10]. As a multidimensional response to the stressors associated with the caregiving experience, caregiving burden can have devastating and long-term effects on family caregivers [11,12].

Researchers have developed a range of technological interventions to assist in reducing the caregiving burden and overcoming the challenges faced by family caregivers [13,14]. These domains where technology can be leveraged include but are not limited to caregiver platforms, caregiver support, care coordination, telehealth or diagnostics and digital care delivery, alternative therapeutics, transitions of care, housing and operations, and end-of-life planning [15,16]. Although technologies can reduce objective burden, some of the physical responsibilities of caring, and the subjective burden by providing carers access to support negotiation for emotional effort entailed in providing care [17], significant challenges and barriers still exist with respect to the use and adoption of technologies [18-21]. For example, technologies aimed at the caregivers of people living with dementia have been perceived as too complex and can create ethical issues such as reduced privacy, data security, and informed consent [18-21]. In addition, systemic shortcomings, such as a lack of awareness and accessibility and insufficient integration with existing health care services, have limited the rate of technology adoption among caregivers [22]. Given these gaps between technology development and adoption, understanding technology perceptions among family caregivers is becoming increasingly important for identifying specific barriers and facilitators that can then be addressed during the technology development process for promoting the technology uptake.

Recently, researchers began to gather technology perceptions from family caregivers [23-26]. These pioneering works have highlighted a variety of feature preferences relating to technology, with all of them reporting a limited use of technologies because of barriers such as a lack of familiarity, awareness, and availability [23-26]. Although these studies set the groundwork by pioneering the examination of family caregivers’ perceptions of technology in general, there has been limited exploration of (1) how sex, gender, and diversity characteristics shape these perceptions or (2) how the perceptions are considered during the technology development process. The sex and gender gap in general technologies has been extensively studied [27,28]; however, the lack of research on technology perception among informal caregivers still exists despite considerable sex and gender differences with respect to well-being as well as psychosocial and overall health [29-32].

A recent systematic search of the literature on caregiving technology identified only a few studies that have assessed informal caregiver needs with respect to technology from the perspectives of sex and gender [23,33-35]. In all of these studies, there were more female caregivers, ranging from 51% [35] to 73.3% [34]. Sex and gender differences were observed in terms of the perceived usefulness of technology [33], willingness to pay for technology [35], and overall attitudes toward technologies [23,34] designed to assist with caregiving. The systematic search found that the included studies highlighted important differences in the preferences and reception of technology among male and female caregivers but had methodological limitations, including small sample sizes [33,34] and a lack of qualitative studies. Moreover, with the rapid pace of technology development, including an increasing availability through web-based and mainstream shopping, reduced cost of off-the-shelf technologies, and shifting caregiver demographics, these results may no longer reflect the latest trends and opinions toward technology. As such, an up-to-date, in-depth exploration of how sex and gender influence the perceptions of caregiving technology among family caregivers and how these perceptions are incorporated in the technology development process is timely.

This qualitative study aims to address these gaps by examining how family caregivers perceive the use of technology in assisting with their caregiving routines; identifying any sex, gender, and diversity factors that shape these perceptions; and how these perceptions and needs are reflected in the current technology development process. We took a qualitative descriptive approach to allow for a detailed description and understanding of the *who, what,* and *where* of technology perceptions, as it relates to caregiving and technology development [36]. By drawing on the general tenets of naturalistic inquiry, which involves the shift from tangible variables to focus on the social constructions of research participants, this study also aims to explore these technology perceptions as a product of characteristics and behaviors that are socially and culturally contingent [36,37].

## Methods

### Participants

Our study sample comprised 16 adults providing care for an adult or older adult family member and 8 technology researchers. Tables 1 and 2 describe the characteristics of the family caregivers and technology researchers, respectively. These individuals were recruited using purposive sampling from the communities in Ontario, Canada, through various organizations, such as the March of Dimes Canada, Disabled Women’s Network Canada, Aging Gracefully across Environments using Technology to Support Wellness, Engagement and Long Life, University of Toronto, University Health Network, and Health Quality Ontario (collectively referred to as *recruitment partners*). The staff of these organizations distributed study emails and flyers to potential participants. In addition, members of the research team (CX and GEKR) delivered presentations about the study to potential participants at meetings organized by our recruitment partners. Interested participants contacted the research coordinator (GEKR) by phone or email, where they received more information about the study.

Participants were eligible if they were aged ≥18 years, able to communicate in English, and unaffected by neurocognitive or physical conditions that might have impeded interviewing. In addition, to be included, family caregivers needed to be taking care of an adult member in their household for the past 12 months for an average of 3 hours or more per week. The inclusion criterion for technology researchers was that they had been involved in projects that developed technologies for assisting with caregiving for the past 12 months.

**Table 1:** The characteristics of family caregivers within this study (N=16).

Characteristics of family caregivers			Values, n (%)
**Age (years)**			
	≤55	9 (56)	
	>55	7 (44)	
**Assigned sex**			
	Male	6 (37)	
	Female	10 (63)	
**Gender**			
	Cisgender man	6 (38)	
	Cisgender woman	9 (56)	
	Gender queer or gender nonconforming	1 (6)	
**Highest level of education**			
	Some postsecondary education or below	4 (25)	
	Trade certificate, bachelor’s degree, or university certificate below bachelor’s degree	8 (50)	
	Advanced degree	4 (25)	
**Ethnicity**			
	European origin	11 (69)	
	Other (Asian, Canadian descent, or Caribbean)	5 (31)	
**Marital status**			
	Never married	5 (31)	
	Married or common law	5 (31)	
	Divorced, separated, or widowed	6 (38)	
**Employment status**			
	Full-time or part-time employment	6 (38)	
	Unemployed, retired, or on disability support	8 (50)	
	Not reported	2 (12)	
**Finances at the end of the month**			
	Just enough to make ends meet	6 (38)	
	Some or more money left over	7 (44)	
	Not reported	3 (18)	
**Length of care provision (years)**			
	1-2	5 (31)	
	3-5	4 (25)	
	≥6	7 (44)	
**Care recipient condition^a^**			
	Musculoskeletal	7 (44)	
	Dementia	5 (31)	
	Cardiopulmonary	8 (50)	
	Psychological	7 (44)	
	Neurological	4 (25)	
	Hepatic, renal, or digestive	5 (31)	
	Other	10 (63)	
**Number of care recipient conditions**			
	≤3	10 (63)	
	≥4	6 (38)	

^a^Overlap due to multiple reported conditions per care recipient.

**Table 2:** The characteristics of technology researchers within the study (N=8).

Characteristics of technology researchers		Values, n (%)
**Age (years)**		
	≤35	5 (63)
	>36	3 (37)
**Assigned sex**		
	Male	3 (37)
	Female	5 (63)
**Gender**		
	Cisgender man	3 (37)
	Cisgender woman	5 (63)
**Highest level of education**		
	Some postsecondary education	1 (13)
	Bachelor’s degree or university certificate below bachelor’s degree	4 (50)
	Advanced degree	3 (37)
**Ethnicity**		
	Chinese	3 (37)
	European origin	2 (25)
	Other (Iranian, Arab, or Canadian descent)	3 (37)
**Occupation**		
	Professor	1 (13)
	Research staff	3 (37)
	Student	2 (25)
	Other	2 (25)
**Work location**		
	Research lab	6 (75)
	Other	2 (25)

### Procedure

Ethics approval was obtained from the University of Toronto, and all participants provided verbal and/or written informed consent for participation and the use of their information.

Semistructured interviews were conducted in person or over the phone with (1) family caregivers to explore their technology perceptions and (2) technology researchers to examine how they incorporate caregivers’ perceptions within the technology development process. Before conducting the interviews, GEKR had no contact with the participants except for scheduling the time and place of their meeting (if applicable). Interviews were conducted in a private room at the University of Toronto, ranged between 45 and 120 minutes, and were recorded using an encrypted digital recorder. Before commencing the interviews, GEKR explained the purpose of the study and the detailed procedure of the interview to the participants. Open-ended questions were first asked to initiate discussion, and probing questions followed to further enrich the conversation. Upon the conclusion of the interview, GEKR collected a demographics questionnaire from the participant. Remuneration was offered in the form of a Can $40 (US $30) gift card delivered via mail, in person, or via email. Field notes were taken by GEKR during and after each interview, which were used as a starting point during our analyses and discussion.

During each caregiver interview, participants were asked about their knowledge of technology related to caregiving; experiences with technology; and how they perceived the influences of sex, gender, and diversity on their technology use. For the interviews, technology was communicated to participants as digital technologies, such as, but not limited to, mobile apps, medications, and smart home technologies. Nonetheless, participants were given the opportunity to explore other technologies that they were aware of or have used within their caregiving context. During each technology researcher interview, participants were asked about their current research; knowledge of sex, gender, and diversity considerations in caregiving technology; and the extent to which these considerations were incorporated in the technology development process. Full versions of the interview guides for caregivers and technology researchers are provided as Multimedia Appendices 1 and 2.

Verbatim transcriptions were outsourced to a data transcription service (NVivo Transcription; QSR International). Each transcript was reviewed by 2 members of the research team (CX and GEKR) to ensure accuracy. To ensure confidentiality, all participants were assigned an alphanumeric code, and any identifying information was removed from the data corpus during the review of the transcripts.

### Data Analysis

All data from the transcripts were coded and analyzed using the framework method [38], which is a form of thematic analysis designed to identify the patterns across a data corpus and describe participants’ experiences and perspectives [39,40]. In addition, thematic analysis has the potential to yield complex and nuanced analyses [39,40]. The framework method comprises the following 7 stages: transcription (CX and GEKR), familiarization with the interview (CX and GEKR), coding (CX and GEKR), the development of a working analytical framework (CX and GEKR), the charting of data into the framework matrix (CX, GEKR, and AS), and data interpretation (CX and AS) [41]. By providing clear steps for following and producing a visually straightforward presentation of patterns and themes, the framework method is helpful for projects with multiple groups of participants, as in our study [41].

NVivo was used to code and manage the coded data. Transcribed interviews were coded independently by CX and GEKR, who noted all caregivers’ perceptions and experiences relating to technology from the caregiver interviews. During the preliminary coding of the initial interviews, the authors uncovered additional significant influences on technology perceptions in addition to sex and gender. Furthermore, with the advancement of sex and gender understandings, a paradigm shift was observed in the interpretation of sex and gender within a more comprehensive framework that prioritizes diversity [42]. As such, the authors examined sex and gender in conjunction with other diversity factors that may shape technology perceptions, including, but not limited to, age, socioeconomic status, personal relationships, ethnicity, and culture.

Given the aim of examining how caregivers’ perceptions of technology are integrated within the technology development process, the authors discussed their coding processes and created an analytical framework based on the collated codes gathered from the transcripts of both technology researchers and caregivers. This allowed the charting of the data onto the developed framework. During the final data interpretation stage, the authors moved beyond collated codes by sorting them into categories and refining these categories into themes. Regular discussions between the authors were held during each step of the data analysis, facilitating further exploration of participants’ responses, discussion of deviant cases, and agreement on recurring themes.

## Results

### Overview

Tables 1 and 2 describe the characteristics of the family caregivers and technology researchers included in this study, respectively. Among family caregivers, approximately 63% (10/16) were females and 56% (9/16) self-identified as cisgender women. The majority of caregivers were of European origin and were aged ≤55 years. Family caregivers within our sample have a diverse range of education levels, length of care provision, marital status, and employment status. Care recipients have a wide range of medical condition types, including, but not limited to, musculoskeletal, cardiopulmonary, and neurological conditions. Among the technology researchers, approximately 63% (5/8) were females and self-identified as cisgender women. Most technology researchers work in a research lab and were aged ≤35 years. Technology researchers within our sample have a diverse range of education levels, ethnicities, and occupations.

Three main themes with subthemes were developed to capture the benefits and challenges of using and adopting technologies for caregiving. The first main theme is that *caregivers see a need for technology in their lives,* and it comprises 3 subthemes: *caregiving is a challenging endeavor, technology is multifaceted*, and *caregiver preferences facilitate technology use.* The second main theme is that *relationships play a vital role in mediating technology uptake,* and it comprises 2 subthemes: *the caregiver-care recipient dynamic shapes technology perceptions* and *caregivers rely on external sources for technology information.* Finally, the third main theme is that *barriers are present in the use and adoption of technology,* and it comprises 2 subthemes: *technology may not be compatible with personal values and abilities* and *technology not tailored toward caregivers lacks adoption.* Finally, the influences of sex, gender, and diversity will be discussed with respect to all 3 main themes.

### Theme 1: Caregivers See a Need for Technology in Their Lives

#### Caregiving is a Challenging Endeavor

Caregivers describe several difficulties in fulfilling their caregiving duties. Specifically, the need to provide care to their care recipient challenges their ability to meet the needs of both themselves and the care recipients. Caregivers report making significant adjustments to their daily routines to provide care. For example, one caregiver notes that he is no longer able to exercise and his life essentially revolves around caring for his mother as well as his own job (CG03). By putting the care recipient’s needs above their own, caregivers face difficulties in striking a balance and often find themselves neglecting their own health despite recognizing the importance of looking after their own well-being. As one caregiver expresses:

Everyone tells me, “Oh you need to look after yourself; if you look after yourself, you can look after her.” Yes, it’s all well said and done.

Caregiver 06

As a result of these changes entailed while providing care, caregivers were both physically and emotionally burdened by the entire caregiving experience. Faced with the stress of having to take on a lot within a very short time, caregivers experience a drop in their quality of life. The health care system further exacerbates the caregivers’ physical burden and is called a *rugged* system that requires a lot of effort to receive assistance. Caregivers highlight a lack of clarity in the information provided and lengthy wait times for services such as home care support.

In addition to the physical difficulties that are experienced, caregiving also puts an emotional drain on caregivers, as CG02 shares:

I worry about her because she cannot. It’s hard for her to defend herself because of the language issue...the memory issue, because she has no power and no credibility. So she’s highly vulnerable, so I feel stuck with that and I also feel, I don’t know, scared for what’s going to come.

Caregiver 02

As CG02 takes on the caregiving role, she is confronted with the uncertainties associated with the ever-changing condition of her care recipient. She expresses the fear of what the future holds, a sentiment shared among several caregivers in the study. For others, the emotional drain takes the form of guilt toward the care recipient. In particular, caregivers develop the perception that they are not doing enough and that there is much more that they could do to help their care recipient.

#### Technology Is Multifaceted

Technology plays a multifaceted role in mediating caregiving challenges. Caregivers describe a wide range of tasks for which they perceive technology to be the most useful. Specifically, caregivers value the convenience of using technology to connect and communicate with loved ones on demand and remotely when the caregivers are away. In a similar vein, caregivers use technologies to obtain up-to-date information and resources, as CG18 shared:

See for me...Googling stuff is my way of finding out information about my father. So, I’ll go on like WebMD, I’ll go on the Mayo Clinic, like things like Diabetes Canada, Health Canada

Caregiver 18

By using the internet, caregivers are able to save time and effort in obtaining information and redirect this time and effort toward taking care of their care recipients. This not only increases their caregiving efficiency but also opens opportunities for caregivers to engage in self-care activities. From browsing social media to using meditation apps, caregivers see technology as a tool to allow them to relax and unwind amid their hectic schedules, which can also be managed using technology. As CG04 notes:

As part of Google Calendar, which I use almost conclusively, you can set up a variety of calendars there: your personal, your work. But yeah, there is one for [my care recipient] in there. I mean, she does not see it because she doesn’t have a computer really. She tells me about an appointment or whatnot, I go right in then and just type that sucker and make sure to follow up.

Caregiver 04

With these internet-based calendars, caregivers can better plan their day and avoid running the risk of missing appointments or scheduling conflicting commitments. Given the need to balance both personal and care recipient needs, these time management tools allow caregivers to appropriately apportion the time between themselves and the care recipient. With regard to care provision, technology has been used to support regular chores and provide a sense of security to caregivers. By enabling caregivers to keep track of care recipients’ health status and whereabouts on a regular basis, the use of technologies, such as wearable devices and home monitoring cameras, has been mentioned by the majority of caregivers as a means of alleviating caregiving burden and providing them with a piece of mind.

#### Caregiver Preferences Facilitate Technology Use

Although technology has its rightful place in helping with caregiving, not all technologies are created equally. Caregivers report looking for specific features and characteristics of the technologies that they are willing to use on a regular basis (Textbox 1). Technologies that do not encapsulate these characteristics are not as well-received. In line with the dominant expectations of technology acceptance and adoption between genders [43,44], a greater proportion of males (within our sample) express a greater appreciation of and a desire to acquire the latest technologies. As one caregiver notes:

I am like an early adopter of technology. Like I always try to be first among people to get technology. I mean when I got my phone like it was just on the market for like three months before I like I went for it....

Caregiver 18

In contrast, female caregivers in our sample tend to gravitate toward technologies that resemble the items that they previously used. As one female caregiver expresses:

I think however we can use make technology, design a technology in a familiar way even if there’s all kinds of fabulous things going on, you know, a little microchip, put in a huge box just to make them feel like they are using something that they remember using in the past.

Caregiver 01

Female caregivers are uncomfortable in acquiring new technologies that are unfamiliar to them, citing reasons such as an inability to unlock the technology’s full potential. In addition, older caregivers (aged >55 years) expressed a desire to have technologies that are packaged in a way that is familiar to them.

**The technology preferences of family caregivers, technology descriptions, and quotes.**

**Accessibility**

- Technology with features that meet specific needs
- Accessibility considerations include hearing, visual, or mobility impairments; communication disorders; and learning disabilities
- Products should include features that speak to these key areas, such as screen readers, speech recognition, adaptive keyboards, and simplified language and instructions.
  “I think if there was like the ability to have...that personal one on one aspect in terms of the development of the products, it might be beneficial, just because...each person’s spinal cord injury, in my experiences, has been very, very different from the others. So if there was like a customized element to it, it would probably make it more attractive to me to want to even invest the money, even if it was costly.” [CG15]

**Bang for the buck**

- Caregivers consider cost effectiveness, affordability, and value for money while making decisions about purchasing technology.
- Devices should be long lasting, with a widespread availability of parts and software updates for older models.
  “I know they got to make money to cover the development of the app.... But still, it’s got to be a reasonable fee. Charging a hundred dollars a year, two hundred dollars a year, is going to make it impossible for some people in certain categories and you’re going to end up with one sector of the population able to use it and another sector unable because they can’t afford it.” [CG12]

**Blast from the past**

- Caregivers feel more comfortable using products that look and operate in a way that is familiar to them or resemble something from their background.
  “It has to be in language because you’re dealing with people who are aging and have some problems with cognition or ability to adapt and to incorporate new technology into their life.... They have to be using something that looks or feels like something from their past.” [CG01]

**Eco-friendly**

- Caregivers cited a preference toward technology that minimizes environmental impact, including products that are recyclable or otherwise reduce carbon footprints.
  “I think if something was eco-friendly it would make a huge difference. I think people would be more inclined to actually get it because they don’t want to...increase their carbon footprint so that that would be important to me at least.” [CG15]

**Latest and greatest**

- Caregivers would like to be aware of the latest technology and be at the forefront of products that can assist with caregiving.
- A consideration for technology adoption includes the availability of consistent upgrades to the software and hardware.
  “...What I’d look for in technology, something where software is constantly being upgraded. Like [my] phone upgrades itself sometimes three times a day every, it’s like a new phone. It is literally like a brand new phone once a day.... So I constantly get the technology. It comes through immediately. And that’s what I would look for: upgrading.” [CG03]

**Multifunctionality**

- Due to their busy lifestyles and multiple responsibilities, caregivers have a strong preference for products that can assist with various caregiving tasks simultaneously.
  “If I was to get the tech, if there was an application my mother could figure out or if there was a caregiver where I could split the screen. Talk to the caregiver on one half of the screen and then the other half still be able to keep a line open for business and whatever I have to do.” [CG03]

**Readily available training**

- Caregivers would feel highly supported with the availability of clear resources that assist with the operation and use of the technology.
- This includes information on how support can be accessed, minimal wait times, and 24-hour availability.
  “Make all kinds of support readily available. If you have an issue at 3:00 p.m., at 3:00 am. Let make there be like a technical support staff that you can talk to. You know, live chat, email, texting.... So there’s always someone readily available to address your needs.” [CG18]

**Seamless operation**

- To better integrate technology into their lives, caregivers prefer products that require minimal user input and interaction.
  “It is like really...non-invasive, easy for her to...use in the sense. It seemed almost like seamless for her, like seamless technology.” [CG06]

### Theme 2: Relationships Play a Vital Role in Mediating Technology Uptake

#### Caregiver-Care Recipient Dynamic Shapes Technology Perceptions

Caregivers report frequently taking the opinions of the care recipient into account when faced with technology decisions. The way technology fits within the caregiver-care recipient space determines how it is being perceived and how likely it is to be adopted within the caregiving context. Caregivers are eager to convey the importance of including the care recipient within the discussion on technology use and uptake. Perceptions toward these devices have been reported by caregivers as being established through a 2-way conversation between themselves and the care recipient. One caregiver taking care of her mother describes the process of acquiring technology to help with caregiving, saying:

I know with families you have different ability levels as it relates to technology, so I’m thinking if I was sharing this responsibility with my mom and we both wanted to input on something, it would probably [have] to be something fairly simplistic.

Caregiver 08

In the caregivers’ experience, they consider not only their own level of technology competency but also that of their care recipients, which, on occasion, takes precedence over their own. This has led to instances where opportunities to enhance caregiving using technology are missed, as one caregiver taking care of her mother notes:

As my mother deteriorated and she was in another city, about like 45 minutes away, I really looked into some technology to sort of bridge that gap because you know – it...could hopefully help me in helping her. Unfortunately, she’s very anti-technology because she was born in that time when you [didn’t] rely on machines, you [relied] on human beings, so it’s been a bit of a struggle to sort of help with the assistance of technology.

Caregiver 04

#### Caregivers Rely on External Sources for Technology Information

When it comes to identifying resources such as technology to assist with caregiving, caregivers report a disconnect between reality and the support that ought to be available. One caregiver taking care of her mother sums up her experience in obtaining support from the health care system:

The health, the community, everything else, you know, all of these supports that were “supposed to be there,” you know, none of them are there. So, you know, all – the whole burden – is come up to me. And so, once they look at it, that I’m there, they’re basically probably, you know, going to say “Oh she’s doing it. So, we really don’t need to provide that service now.”

Caregiver 06

The process of navigating the health care system is perceived as a burdensome process that lasts for a long time for the caregivers. There are many obstacles in the process of obtaining help, which is mainly caused by a lack of communication between different health care entities. Owing to a lack of support from the public health infrastructure, caregivers turn to their peers for technology information. They highlight the importance of using their social networks and connections as an avenue to gain awareness of technology and support them within their roles. A case in point is CG07, a caregiver who turns to her son, a former computer engineer, for technology-related information:

We also, well, you know, we have a son who is an electrician. He is actually an industrial electrician. Now, I would ask him to because he was a computer engineer before. Before he did that. So once, you know, he is a computer [person].

Caregiver 07

By approaching these alternative information sources, caregivers are introduced to various technologies through word of mouth and experiences of peers who are in similar caregiving situations. Given similar backgrounds and high levels of rapport, caregivers see these sources as credible and well intentioned, which represents a stark contrast to their expressions of disappointment and doubts with government support and assistance.

### Theme 3: Barriers are Present in the Use and Adoption of Technology

#### Technology Is Not Compatible With Personal Values and Abilities

Although caregivers recognize the benefits that technology can bring to their lives, they continue to face a wide range of challenges related to its use, which in turn limits its adoption. Most caregivers do not have extensive knowledge of the types of technology, skills to fully take advantage of its potential, and the ability to troubleshoot any technical issues. When asked to describe his perceptions of the barriers to technology adoption, one caregiver noted:

The average person does not have the idea that it exists, but if they have any ideas, they have no way of contact. There’s no one to contact to see if that could happen. You know, to see if it’s even possible to ask if it’s out there. I mean there’s a lot of things that are out there. People aren’t aware of because they don’t know [whom] to call.

Caregiver 07

As much as technology has evolved and proliferated over the last few years, there remains an information gap between technology development and its intended users. Caregivers do not have the time and energy to actively seek out available technology, especially when there is a lack of a centralized information source or resource for such information. As previously mentioned, much of their technology awareness is attributed to their interaction with peers, friends, and family. Therefore, for a technology to be adopted by caregivers, its introduction ought to involve the caregivers’ social and support networks, such as their physicians, nurses, other allied health professionals, and support organizations. Alternatively, greater emphasis can be placed on the development and promotion of centralized information centers for technology where caregivers can seek out information on available technologies. However, difficulties with technology go beyond the acquisition process. As one caregiver describes her experience with technology:

When a person is under stress, as we know, they’re not able to function as if they didn’t have any stress. So, you couple...the illness and then add to it a stress level which leads to a panic mode. And you’re unable to do a lot of very simple operations. You get frustrated, annoyed, upset, all of the above, and nothing works very well.

Caregiver 09

The worries and exhaustion of caring tasks, the stress of being the caregiver, and the management of the care recipient’s illness come together and interfere with the ability of caregivers to operate technology, which has been described as complicated, unintuitive, and difficult to navigate. This creates even more stress and frustration as caregivers struggle to make sense of their devices. As such, a vicious circle is created, which, as CG09 notes, makes caregivers feel angry and annoyed at the technology. In addition, caregivers are also concerned about the security and privacy of their data while using certain technologies. They are reluctant to share information about themselves and their care recipients without knowing who will have access and how their data will be used.

Given these challenges in gaining awareness and using technology, many caregivers return to previously established caregiving routines that do not involve technology, as they are not only familiar with these tasks but also able to preserve a level of in-person interaction, which has been gradually eroded with the introduction of technology. As one caregiver describes:

You reach out to try to find help. And in real life there really isn’t a whole lot of support out there. So yes, you have to turn to technology...which is such an anonymous support and doesn’t at the end of the day give you any kind of “Oh good job”, no pats on the back.

Caregiver 05

From the caregivers’ perspectives, technology is limited in providing feedback and validation of their actions. In contrast to the traditional forms of caregiving support, such as peer groups, caregivers perceive that they are unlikely to experience the same level of interpersonal connections through technology use. As such, caregivers will only accept these technologies in situations where they have no other choice, such as in rural areas or when they are at a distance from their care recipient.

#### Technology Is Not Appropriately Tailored Toward End Users

Caregivers note that the current technology has not been designed with their needs in mind. As one caregiver looking after her mother expresses:

If they have used someone with the lived experience, they would have designed all of this technology there. People who don’t have the lived experience or loved one[s] with the lived experience – so they’re doing this in a vacuum

Caregiver 01

In addition to lacking a basic understanding of the caregiving experience and perspective, caregivers also perceive technology development to be an isolated process that does not consider the diverse needs and preferences across the caregiving population. In particular, caregivers are concerned about the lack of accommodation in language and cognitive abilities, which can vary widely across individuals. As one caregiver looking after her mother comments:

It has to be in [their] language because you’re dealing with people who are aging and have some problems with cognition or ability to adapt and to incorporate new technology into their life.

Caregiver 01

These sentiments are in stark contrast with the perspectives of technology researchers, who highlight the importance of involving caregivers during the technology development process. As such, the conflicting remarks represent a gap between the perceptions of technology researchers during their development process and the reality caregivers are facing.

#### Technology Researchers’ Perspectives

For technology researchers, building rapport and establishing a relationship with caregivers have been highlighted as important steps in the technology development process. Technology researchers recognize the need to involve caregivers during the development process through consultations and workshops. As a technology researcher working in the field of caregiving technologies for the past 5 years describes:

I involve the people I’m building the technology for in the design and development the whole ways through, like ideation, prototyping, feedback on early prototypes and then efficacy. It’s not just building in a vacuum.

Technology Developer 05

By involving caregivers in the development process, technology researchers have been able to build a level of empathy with these end users. Not only are technology researchers able to gain a firsthand understanding of the needs and feedback of caregivers, but they also expressed a greater motivation to make a positive impact on their lives by building technologies that fully address their needs and preferences. Furthermore, technology researchers report various efforts they have begun making to take a variety of relevant demographic and sociocultural factors of caregivers into consideration within their own work as appropriate. These factors span physical characteristics, such as ability and body type; socioeconomic indicators, such as education and rurality; and cultural factors, such as family background and language. For example, one technology researcher working on an app that monitors caregiver posture comments:

That should do. Definitely I think weight is one thing that would affect it. Because generally to people who are thinner would be easier to bend so they just go and bend whenever they wanted. But the people who are a bit more heavy, and have some more weight generally tend to bend less because the posture loads are bigger.

Technology Developer 01

By recognizing the diverse range of factors that influence technology use and perceptions, technology researchers have taken a first step in ensuring that the needs of caregivers are being better addressed through technology. Nonetheless, despite acknowledging and incorporating the diverse characteristics among caregivers within their work, most technology researchers within our sample continue to hold assumptions about caregivers’ technology needs and preferences. Technology researchers’ perspectives tend toward generalizations, grouping caregivers into stereotypical buckets that may not reflect the broad spectrum of needs across diverse populations. For example, when asked about sex and gender influences on technology and caregivers, a technology researcher commented:

When I’m trying to imagine a community of caregivers, I would think that it’s much easier to promote a device across the female population as they probably tend to share more among each other. And if they come up with a good device, [they’re] probably gonna tell other people, or if they need support, they are going to ask. Which is like not as good among the male population. And they’re probably not going to be that much sharing and then, at the same time, not that much caring about their fellow male care providers.

Technology Developer 07

Beyond the conceptual misunderstandings, technology researchers also face challenges in incorporating diversity within their current work, which has been attributed to budgetary constraints. When asked to describe his research process, one technology researcher commented:

Yeah, funding is always a problem. Yes, I mean I think that’s true of everyone right. Like even bigger corporations that have a lot more flexibility in terms of that, they still have a budget to run and they still have a quarter leg and everything else, so you know if money was infinite, then things are pretty much infinitely possible.

Technology Developer 05

Owing to a lack of funding and time constraints, technology researchers are often limited in their ability to recruit diverse participants in their development process. In addition, the sheer range of diversity factors, including, but not limited to, age, socioeconomic status, education level, and geographical location, requires the collection of a large number of data points and variables as well as expertise in conducting a sex- and gender-based analysis, both of which add complexity to the already challenging endeavor of technology development.

## Discussion

### Principal Findings

In this first-of-its-kind study to our knowledge that included both family caregivers and technology researchers, we analyzed interviews to gain a deeper understanding of technology perceptions among caregivers as well as whether these preferences are being incorporated within the technology development process. Through our analysis, it is apparent that caregivers perceive technology as a double-edged sword. Caregivers see technology as a valuable addition to their caregiving routines by opening a range of opportunities for them to enhance their care provision and reduce their caregiving burden. However, caregivers remain wary of the limitations and complications technology use may bring; it is associated with privacy concerns with personal health information; a lack of personalized feedback; and above all, an added frustration when things go wrong. With respect to sex, gender, and diversity, it is recognized that although sex and gender differences are explicit in technology preferences among caregivers, characteristics such as physical and cognitive abilities as well as caregiver-care recipient relationships have an influence on other aspects of technology perceptions, including barriers and uptake.

We demonstrated how caregivers’ needs for technology ranged across caregiving contexts. In line with previous literature [7-10], caregivers described caregiving experience as stressful and filled with numerous challenges. Faced with these significant physical, psychological, emotional, and financial burdens, caregivers have sought support from various sources, including health care and governmental organizations. For some caregivers, these groups have facilitated access to resources, including technology to assist with their caregiving tasks. However, for many other caregivers, navigating these organizations proved to be a challenge. Hence, they turn to alternative sources of informal support, which include the use of technology. As such, the findings reveal a need for technology among caregivers driven by not only caregiving burden [45] but also the lack of well-advertised and accessible support within the health care system. This presents a pressing need for the current health care systems to implement changes to improve their reach and, most importantly, streamline their processes for caregivers looking to access support, a move that can be facilitated by using technology.

Similar to a previous study on technology adoption conducted with older adults [46], caregivers have identified a wide range of features and characteristics that they are looking for while considering technology. These factors include not only the physical function and design but also the acquisition process and the after-sales support. Given the diverse range of factors to consider, these preferences highlight the multifaceted nature of caregivers’ technology adoption. Further adding complexity is the caregivers’ sociocultural backgrounds, which play a significant role in informing their perspectives toward technology. Unlike younger caregivers, we found that older caregivers in our sample tended to express a desire for technologies designed and operated in a way that is familiar to them. A similar observation was also found with gender, with female caregivers in our sample preferring familiar technology. These findings are congruent with previous conceptual work on technology adoption across the general population, which have highlighted age and gender as important factors that shape technology acceptance [47-49].

In addition, our study uncovered the influence of caregivers’ external environment in determining technology awareness and uptake. Caregiving is rarely done in isolation, and caregivers recognize the need to consider the views and opinions of their care recipient while considering technology. Such a collaborative spirit has also been reflected in the caregivers’ interactions with their informal networks, particularly interactions with peers, family, and friends. Technology has become an integral part of caregivers’ conversations with these individuals. As such, these social networks influence caregivers’ attitudes toward technology. Specifically, caregivers with a tech-savvy family and friends often described technology in a more favorable tone. In contrast, some caregivers with little or no exposure to technology in their social circles paint a bleaker picture of potential technologies to assist them in their care routines. Given the prevalence of obtaining technology-related information through word of mouth among caregivers, it may be worthwhile to explore alternative methods of technology dissemination, such as social media and caregiving support groups. Information about technology could also be made available by health and social care and nongovernmental organizations providing care and support to people living with various conditions that family caregivers support. For example, the Alzheimer’s Society in the Durham Region of Ontario has a selection of currently available technologies that clients can test. These clients can also ask questions to an informed staff member.

Our study also highlighted the existing barriers caregivers faced while acquiring technologies to help with caregiving. Given that most of the caregivers in our study were older adults, it is expected that their views would be congruent with findings from recent work on technology adoption among the general older adult population [50-52]. Specifically, these barriers are characterized by concerns regarding technology literacy, user-friendliness, accessibility, and privacy. Caregivers in this study tend to associate technology adoption with a lack of ability to understand or operate them. As a result, it creates a source of frustration during their interactions with technology, which is further exacerbated by the unintuitive and hard-to-navigate interfaces of both hardware and software. As these older caregivers may not be introduced to technologies until later in their professional careers, they may have a lower level of technology literacy that impedes their ability to adapt or welcome new technologies within their lives. Hence, they may have felt more discomfort by changing their routines and embracing foreign technology, especially if its use requires a substantial learning curve. In addition, the physical and cognitive changes associated with the aging process have limited the accessibility of many technologies. Caregivers often find that technologies are not designed to take account of their physical and cognitive abilities, especially in the area of visual and audio enhancements, which have been suggested to be inadequate or poorly designed for common uses [50]. Finally, caregivers are hesitant to surrender their personal data to connected technologies. Such a mistrust in the data handling processes can be a significant obstacle in the development and introduction of technologies driven by big data analyses.

Given the diverse spectrum of technology needs and preferences among caregivers (Textbox 1), technology researchers need to take steps to better understand and address these when developing solutions aimed at caregivers. Although the researchers report that they recognize the importance of involving and connecting with caregivers to solicit their perspectives during the technology development process, in practice this seems not to be done in any systematic way. The technology researchers in this study have reported several internal and external obstacles that limit their ability to understand caregiver perspectives. For some researchers, the assumptions and stereotypes they make about caregivers and their roles occlude the perceived importance of approaching individuals with actual lived experiences. By holding on to personal opinions without corroborating research evidence, technology researchers have overlooked the importance of understanding the diverse needs within these groups. Being interrelated, sex and gender are not binary constructs but rather on a continuum, which necessitates greater attention and intricacy in disentangling the influence of both concepts in technology perceptions. As social identities, these interrelated concepts also intersect with other social identities, including, but not limited to, race, culture, and age, to create unique personas and perceptions toward technology across the caregiving population. Although they appreciate the value added by caregivers to the product, these researchers struggle to engage the caregivers of diverse backgrounds during the development process. Factors such as funding, time, data availability, and expertise have all been cited as obstacles in the process of incorporating diversity within technology development.

To help overcome these challenges, alternative design approaches, such as user-centered, participatory, and experience design, have been proposed [53]. These approaches provide models for the involvement of end users throughout the development process and, in many instances, place the focus on the user rather than the product [53,54]. However, as general approaches are applied across a wide range of product developments, they may not help in navigating the nuances of technology within the caregiving context and provide guidance on the incorporation of sex, gender, and diversity considerations, which are areas where more training and expertise are needed. As such, future technology projects can consider the assembly of multidisciplinary research teams that integrate members with experience working with caregivers and studying their perspectives from a diversity perspective.

### Strengths and Limitations

To our knowledge, this is a pioneering work in the field of technology perceptions across family caregivers using qualitative research methods. By including the caregivers of persons with various conditions and needs, we have captured and drawn attention to the diverse technology experiences and perspectives across caregiving contexts. With one of the largest samples for such a qualitative study, the findings highlight the multifaceted role technology can play in aiding caregiving while highlighting the drawbacks of these technologies perceived by caregivers. Furthermore, by including technology researchers in the study, we gathered a more holistic understanding of technology in caregiving from its initial development to eventual uptake by caregivers. In particular, the findings reveal the diversity of caregivers’ technology needs and perspectives that will need to be addressed during the technology development process.

Limitations of the study include the rather small sample sizes of technology researchers, which is limited in part due to our criteria for a focus on technologies relevant to caregiving. Future research can be conducted with a greater number of technology researchers to better capture the technology development process across a wider range of technology fields. Although our work includes both caregivers and technology researchers, the 2 main players within the field of caregiving and technology, we recognize that there are other stakeholders such as care recipients, health care providers, and policy makers. As such, future work should consider the perspectives of these stakeholders and explore the interactions between them. This is very relevant for exploring issues related to access to technology, including financial barriers, that caregivers, with their special needs to support their caregiving routines, face. Finally, it is recognized that the findings may not be generalizable to the entire caregiving population. Hence, it is important for technology researchers to engage their target audiences to learn more about their specific needs and view these results as a guide to the range of worthwhile factors to consider during the development process.

### Conclusions

Family caregivers are turning toward technology to receive assistance in managing the demands of providing care at home. Technology has been helpful in a wide range of caregiving apps; however, there remain several barriers and unmet needs related to its use and uptake. As such, caregivers need support through the course of technology adoption. To provide this support, technology researchers ought to move beyond the current practices of technology development to gather greater knowledge and awareness of caregivers’ diverse needs and preferences. Future work should focus on developing tools and resources for technology researchers to support a greater engagement with diverse caregivers such that their input can be used to develop products that better address their needs.

## Appendix

Multimedia Appendix 1 Semistructured interview guide for informal caregivers.

Multimedia Appendix 2 Semistructured interview guide for technology.
