# Supplementary material for: Perceptions of Digital Technology Experiences and Development Among Family Caregivers and Technology Researchers: Qualitative Study
Source: JMIR Form Res. 2022 Jan 28;6(1):e19967. doi: 10.2196/19967 (PMC8838597; doi:10.2196/19967)
Supplement: Multimedia Appendix 1 [file formative_v6i1e19967_app1.docx]

**Appendix 1**

**Semi-structured Interview Guide for Informal Caregivers**

*Introduction*

Hi, I’m a research coordinator here at the University of Toronto. My team and I are trying to better understand the needs and preferences of caregivers when it comes to digital technologies. I want to start off by thanking you for agreeing to take part in our study, and sharing your thoughts with me. I want you to know that this is a safe and judgment free space and that everything you share with me will be kept anonymous. No one will know that these recordings are associated with you specifically. In order to make sure we are getting the most accurate information it is important to be open and honest about your own personal experiences, however if you feel uncomfortable with answering any of the questions, you may choose not to respond to the question. Before we get started I just want to confirm that it is ok for me to record our conversation. But just to assure you again, it will be kept anonymous.

So today, I’m going to ask you some questions related to your perspectives on the use of digital technologies. A few examples of digital technologies may include but are not limited to mobile applications, medication reminders, and smart home technologies. I also want to know whether gender plays a role in technology usage.

So let me explain a little bit more how the interview will go:

A – I’m going to ask you about your knowledge of digital technologies as it relates to caregiving.

B – Then, I will ask you about your daily routines and your caregiving responsibilities

C – I would then like you to think about how digital technologies might help or hinder a caregiver’s ability to care for their recipient.

D – After that, I would like you to spend a few moments thinking about gender and biological sex, and how it might relate to the digital technologies we choose to use on a daily basis.

E – Finally, I would like you to talk about why you decided to partake in this project and what you would like to see come out of it.

Do you have any questions before we begin?

***Part A***

**What is your understanding of digital technology in the context of caregiving?**

Now, I would like you to clarify some things for me:

1. **Can you explain what these technologies are [based on what participants have mentioned].**

Prompts:

1. What are they called?
2. What are they used for specifically?
3. **How did you find out about these technologies?**

Prompts:

1. Are there others forms in which people could learn about these or other technologies that you know of but maybe didn’t utilize yet?
2. **[If no knowledge on digital technology for caregivers] how come?**

That’s very helpful, thank you. I would like to learn more about your caregiving routines.

***Part B***

**What time do you start your day and what does a typical day look like for you before taking on the caregiving role?**

1. **Could you please describe some if any social or family activities you were doing before becoming a caregiver which you’re not able to do now?**
2. **Could you please share how you have been able to adopt new interests or activities, since becoming a caregiver?**
3. **How do you think digital technologies can help in enabling you to re-engage in activities that you are not able to partake in after becoming a caregiver?**
4. **Are there any devices in particular which might enable your ability to take part in previously enjoyed activities?**

Thank you for sharing that information. I would now like to understand your personal experiences with digital technologies.

***Part C***

**How have you used digital technologies to assist with your caregiving responsibilities?**

1. **Tell me about the technology you use.**
2. **How did you purchase and learn to use these digital technologies?**

Prompts:

1. (if you sense frustration ask) How did you feel in the process of purchasing and learning to use these technologies?
2. Is there something you would change in the process?
3. Is there a particular median that worked best for you when looking into purchasing and researching technologies to purchase?
4. How has this device helped or hindered your ability to take care of your care recipient?
5. Tell me about how it has improved/hindered the relationship you have with your care recipient? With others, such as friends, family members.
6. Tell me about how it has improved/ hindered access to community supports.
7. Tell me about how it has improved/hindered your care recipient’s ability to participate in self-care tasks (e.g., bathing, dressing, brushing their teeth).
8. Tell me about how it has improved/hindered your care recipient’s ability to enjoy leisure activities.
9. Tell me about how it has improved/hindered your care recipient’s ability to engage in productive activities (e.g., volunteer, paid work).
10. Tell me about how digital technologies have improved your life.
11. Tell me about any barriers to using digital technologies. (you might probe on time to learn, confidence with learning, finances, religious/culture beliefs)
12. [If they do not use any technologies] Why have you not used technologies?

Possible Follow up questions:

1. What do you feel is holding you back from using digital technology to assist you with your caregiving role?
2. What can be done to improve access to make it more feasible for you?
3. (if they say they don’t need them or find them useless) Please tell me more, can you give me a particular example of where this might arise?

It’s evident that these specific technologies (list them) have helped in taking care of yourself and your caregiving responsibilities.

***Part D***

As you know, a large part of this project focuses on biological sex and gender. I would like to gain a better idea of what your personal understanding is of biological sex and gender and how this relates to caregiving roles and responsibilities.

**What is your understanding of gender and biological sex?**

[If people are unsure, provide a basic definition, such as biological sex is the sex you are assigned at birth and it is related to your chromosomes, hormones, reproductive organs. Whereas gender is how someone identifies and it’s something that is ascribed to each of us at birth in relation to our bodies. Gender is a social category – it contains the roles, dress, behaviour, and expression expected of a person based on the category given at birth. For example, the idea that women are nurturing and compassionate, whereas men are stoic and unemotional]

**In the context of caregiving and from your own personal observations, how does being a woman or [man] define and describe your role, identity, and expectations as a caregiver.**

1. **What is masculinity and femininity?**
2. **How does a caregiving role impact the way you define your masculinity/femininity?**
3. **Please describe any situations in your life where women [or men or individuals outside of the binary dichotomy] are at a disadvantage when they assume a caregiving role.**
4. **Please describe any situations in your life where women [or men or individuals outside of the binary dichotomy] are at an advantage when they assume a caregiving role?**
5. **How has your daily life been (e.g., family life, social life, work life) positively or negatively affected in assuming the caregiving role?**
6. **How do digital technologies help/hinder women from taking on a caregiver role? [How do digital technologies help/hinder men from taking on a caregiver role?]**
7. **How do you feel about your role as a caregiver?**

***Part E***

Thank you very much for all of your wonderful insights! To conclude, let’s talk about why you decided to partake in this research and what you would like to see come from it.

**What were your primary motivations to partake in this research?**

1. **Did you decide to participate in this project or were you encouraged by someone?**
2. **What would you like to see come from this research?**
3. **What would be some good ways to promote and increase awareness of digital technologies to assist caregivers?**
4. **What would be the best way to present the educational materials? At what location? Who should present the material? What time? How can we promote learning on the topic of digital technologies for you?**
