# Supplementary material for: Perceptions of Digital Technology Experiences and Development Among Family Caregivers and Technology Researchers: Qualitative Study
Source: JMIR Form Res. 2022 Jan 28;6(1):e19967. doi: 10.2196/19967 (PMC8838597; doi:10.2196/19967)
Supplement: Multimedia Appendix 2 [file formative_v6i1e19967_app2.docx]

**Appendix 2**

**Semi-structured Interview Guide for Technology Researchers**

*Introduction*

Hi my name is XX, I’m a research coordinator here at the XXX. My team and I are trying to better understand the perception of biological sex and gender in the development of digital technologies for caregiving among technology researchers. I want to start off by thanking you for agreeing to take part in our study, and sharing your thoughts with me. I want you to know that this is a safe and judgment free space and that everything you share with me will be kept anonymous. No one will know that these recordings are associated with you specifically. In order to make sure we are getting the most accurate information it is important to be open and honest about your own personal experiences, however if you feel uncomfortable with answering any of the questions, you may choose not to respond to the question. Before we get started I just want to confirm that it is ok for me to record our conversation. But just to assure you again, it will be kept anonymous.

Today, I’m going to ask you some questions related to your perspectives on the development of digital technologies. A few examples include mobile applications, medication reminders, and smart home technologies. I also want to know whether sex and/or gender play a role in research, design and development of digital technologies.

So let me explain a little bit more how the interview will go:

A – I’m going to ask you about your knowledge of digital technologies as it relates to caregiving.

B – Then, I would like you to spend a few moments thinking about gender and biological sex, and how it might influence the research and development of these digital technologies.

C – After that, I would like you to think about how we can best promote the uptake of digital technologies among informal caregivers.

D – Finally, I would like you to talk about why you decided to partake in this project and what you would like to see come out of it.

Do you have any questions before we begin?

***Part A***

**Tell me about your research in digital technologies for caregiving.**

Now, I would like you to clarify some things for me:

1. **What are some of the technologies that you and your team are currently developing?**
2. **Can you explain what these technologies are and how they help with caregiving?**
3. **What is the rationale behind the development?**

***Part B***

That’s very helpful. As you know, a large part of this project focuses on biological sex and gender. I would like to gain a better idea of what your personal understanding is of biological sex and gender and how this relates to the research and development of these digital technologies.

**What is your understanding of gender and biological sex?**

[If people are unsure, provide a basic definition, such as biological sex is the sex you are assigned at birth and it is related to your chromosomes, hormones, reproductive organs. Whereas gender is how someone identifies and it’s something that is ascribed to each of us at birth in relation to our bodies. Gender is a social category – it contains the roles, dress, behaviour, and expression expected of a person based on the category given at birth. For example, the idea that women are nurturing and compassionate, whereas men are stoic and unemotional]

**In the context of technology and from your own research experience, how do you incorporate sex and/or gender considerations in the research and development process?**

1. **What is masculinity and femininity?**
2. **Have you experienced or foresee any issue(s) with technology usage that can be potentially attributed to sex and/or gender?**
3. **What are some steps that you have taken in your research to account for potential sex or gender differences among the users of the digital technologies under development?**
4. **What do you think are some of the barriers and facilitators of incorporating sex and gender considerations in the development of digital technologies for caregivers?**
   - ***Probes:* Funding? Feasibility? Regulations? Lack of research?**
5. **Tell me about other factors that you and your team have considered in the research and development process?**
   - ***Probes:* Time to learn, confidence with learning, finances, religious/culture beliefs**

Thank you for sharing that information. I would now like to understand your perspectives on the promotion of digital technologies among informal caregivers.

***Part C***

**How do you think we can better promote the utilization of developed technologies among end users?**

1. **What are some initiatives that your team has embarked on to promote the developed technologies among end users?**

Prompts: Knowledge translation? Presentations? Roadshows? Workshops?

1. **What would be the best way to present the educational materials? At what location? Who should present the material? What time?**
2. **How do you think sex and gender has an influence on the effectiveness of initiatives promoting digital technologies to assist with caregiving?**

***Part D***

Thank you very much for all of your wonderful insights! To conclude, let’s talk about why you decided to partake in this research and what you would like to see come from it.

**What were your primary motivations to partake in this research?**

1. **Were these your own decisions to partake in this research? By whom?**
2. **What would you like to see come from this research?**
